# Remission of Clozapine-Induced Hepatotoxicity Following a Switch to Olanzapine Augmented with Haloperidol: A Case Report and Literature Review

**DOI:** 10.7759/cureus.57341

**Published:** 2024-03-31

**Authors:** Ayodele Atolagbe, Stanley Nkemjika

**Affiliations:** 1 Psychiatry and Behavioral Sciences, Kingsbrook Jewish Medical Center, Brooklyn, USA; 2 Population Health Sciences, Georgia State University School of Public Health, Atlanta, USA; 3 Psychiatry and Behavioral Sciences, Interfaith Medical Center, Brooklyn, USA

**Keywords:** drug-induced liver injury, hepatotoxicity, olanzapine, side effects, clozapine

## Abstract

Clozapine is an effective medication for treatment-resistant schizophrenia, and it has been associated with well-documented side effects that limit its use. Clozapine-induced hepatotoxicity is a less-known complication of clozapine therapy. The literature is unclear about the psychopharmacologic options available following clozapine cessation on account of liver toxicity. We present a patient with clinical symptomatology in keeping with clozapine-induced hepatotoxicity who achieved full recovery following clozapine cessation and conservative medical management. Her psychiatric symptomatology was successfully managed with oral olanzapine augmented with haloperidol without recurrence of psychosis or liver toxicity.

## Introduction

While a transient initial elevation in liver enzymes is common on initiation of clozapine, clozapine-induced hepatotoxicity is a rare occurrence, and a high index of suspicion is needed for its diagnosis and management [1,2]. Drug-induced liver injury is an important cause of liver damage in Western countries and clozapine-induced hepatotoxicity falls under this classification [2]. The onset and severity of liver toxicity secondary to clozapine are variable, unpredictable, and dependent on factors such as age, race, and co-morbid liver disease [2-4]. We present a patient with a diagnosis of treatment-resistant schizophrenia who developed hepatotoxicity while on clozapine monotherapy. Her medical symptoms resolved with conservative care while her presenting psychotic symptoms were successfully managed with olanzapine-haloperidol combination therapy.

## Case presentation

A 48-year-old African American woman with a diagnosis of treatment-refractory schizophrenia presented at the outpatient psychiatry clinic for a routine clinic visit with chills, malaise, loss of appetite, and body weakness ongoing for a few days' duration. Her only medication at presentation was clozapine 100 mg in the morning and 200 mg at bedtime. Her psychiatric symptoms remained at baseline characterized by chronic non-distressing auditory hallucinations. There was no over-the-counter supplement use or illicit substance use.

Her most recent psychiatric hospitalization was six months prior on account of an exacerbation of psychosis. She had presented with auditory hallucinations, aggressive behavior, and paranoid delusions which had failed to respond to first- and secondary-generation antipsychotics. Clozapine monotherapy had resulted in significant resolution of her symptoms, and she was discharged and managed in the outpatient clinic with oral clozapine 100 mg in the morning and 200 mg at bedtime. 

At the current presentation, she was noted to have scleral jaundice, and right upper quadrant tenderness was noted on abdominal examination. Other physical examinations were unremarkable. Her BMI was 36.1, vitals were normal, and urine toxicology was unremarkable. Laboratory values revealed a normal complete blood count and normal serum electrolytes. Liver enzymes at her most recent psychiatric hospitalization and discharge six months prior to presentation were normal. Serum troponin was 0.01 mg/dl. Serum lactate was normal, acetaminophen level was undetectable, and serum creatinine kinase was normal. EKG showed non-specific ST changes and a QT duration of 389 milliseconds.

Serum electrolytes were normal. White cell count was normal without eosinophilia while her liver enzymes were markedly elevated with a serum aspartate transaminase (AST) of 276 u/L, alanine transaminase (ALT) of 145 u/L, serum alkaline phosphatase (ALP) of 147 u/L, and total bilirubin of 3.7mg/dl; serum clozapine was 215 ng/ml and serum norclozapine was 139 ng/ml (Table 1).

**Table 1:** Lab Values on Admission *Clozapine is metabolized to norclozapine and clozapine-N-oxide. Clozapine concentration range between 100ng/ml to 700mg/ml may correlate with clinical response; non-responsiveness may also occur within this range. For refractory schizophrenia, clozapine concentrations greater than 350 ng/mL are suggested to achieve a therapeutic response. BUN: blood urea nitrogen; eGFR: estimated glomerular filtration rate; A/G: albumin/globulin; INR: international normalised ratio; ESR: erythrocyte sedimentation rate; Hb: hemoglobin; HCT: hematocrit; MCV: mean corpuscular volume; MCH: mean corpuscular hemoglobin; MCHC: mean corpuscular hemoglobin concentration; RDW:  red cell distribution width; MPV: mean platelet volume

Comprehensive Metabolic Panel	Value	Reference range
Sodium	137	136-145 mmol/L
Potassium	4.4	3.5-5.1 mmol/L
Chloride	99	98 - 107 mmol/L
BUN	22	7 - 25 mg/dl
Creatinine	1.0	0.5-1.2 mg/dl
BUN/Creatinine ratio	22	8-28
Glucose	89	65-99 mg/dl
Carbon dioxide	26.0	19-32 Meq/L
Calcium	9.3	8.6-10.5 mg/dl
Protein (total)	7.2	6.0-8.3 g/dl
Albumin	4.0	3.5-5.7 g/dl
Alkaline phosphatase (ALP)	147	34-104 U/L
Alanine transaminase (ALT)	145	7-52 U/L
Aspartate aminotransferase (AST)	276	11-39 U/L
Total bilirubin	3.7	0.2-1.0 mg/dl
Direct bilirubin	1.1	0.0-0.3 g/dl
Globulin	3.2	1.8-4.0 g/dl
A/G ratio	1.2	0.8-2.7
eGFR	140	<60 ml/min
Acetaminophen level	6	5-20 mcg/ml
Lactic acid	1.1	0.5-2.2mmol/L
INR	0.9	0.8-1.1 seconds
ESR	43	0-30
Complete Blood Count	Value	Reference Range
White Cell Count	5.9	4.2-11.8 x 10^6/uL
RBC	4.6	3.8 -5.0 x10^6/uL
Hb	13.1	11.3 - 14.9 g/dl
HCT	39.8	34.0 - 44.3 %
MCV	75.4	80.8-97.4 fl
MCH	24.9	26.6-33.0 pg
MCHC	33.0	32.0 - 34.9 g/dl
RDW	15.1	11.8 -15.5 %
Platelet	211	147-365x10^3/uL
MPV	10.0	6.0-12.0 fL
Automated Differential	Value	Reference range
Segmented (%)	51.4	43.7-73.5 %
Segmented (n)	3.0	1.9-7.5x 10^3/uL
Lymphocyte (%)	38.1	17.9-45.1 %
Lymphocyte (n)	2.2	1.0-4.4 x10^3/uL
Monocyte (%)	7.7	3.8-10.0%
Monocyte (n)	0.5	0.2-0.9 x10^3/uL
Eosinophil (%)	2.0	0.0-6.1%
Eosinophil (n)	0.1	0.0-0.5 x10^3/uL
Basophil (%)	0.8	0.0-1.3 %
Basophil (n)	0.0	0.0-0.1 x10^3/uL
Reticulocyte count	1.15	0.29 - 2.38 %
Clozapine (quantitative)*	215	ng/ml
Norclozapine (quantitative)*	139	ng/ml
Clozapine-N-oxide	<100	Ng/ml
Total clozapine and metabolites	354	ng/ml

**Figure 1:**
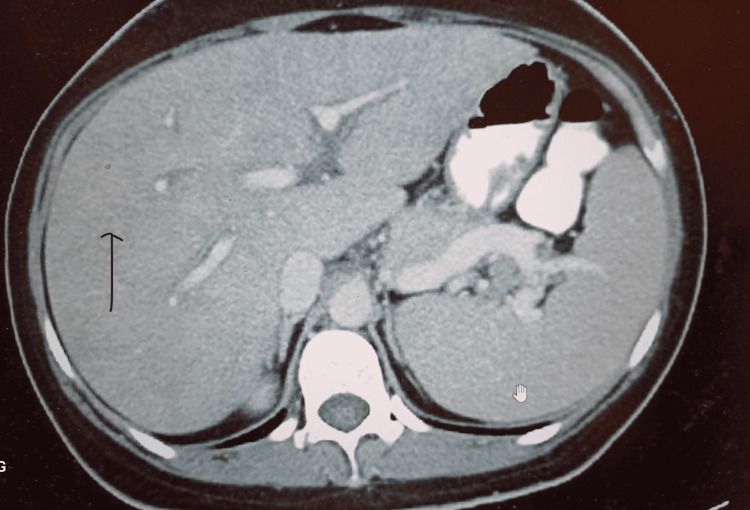
Contrast-enhanced abdominal CT of the patient showing a diffusely enlarged liver without lesions or ascites.

Hepatitis A, B, and C serology were unremarkable, and autoimmune workup was negative for autoimmune markers. The patient was admitted to the inpatient medical floor for further workup and observation. Clozapine was discontinued, and intravenous dextrose and saline fluids were administered. A progressive daily downward trend of liver enzymes was observed with AST, ALT, bilirubin, and ALP at half the initial presenting values at the end of the first week of admission.

She reported increased auditory hallucinations during the course of her medical care and staff had noted a progressive worsening agitation, aggression, and disorganized behavior. She declined clozapine rechallenge but agreed to oral olanzapine at bedtime. The oral olanzapine dose was increased from 5 mg to 10 mg and subsequently to 20 mg at bedtime at the end of the second week of admission; however, she continued to report distressing auditory hallucinations and endorse bizarre delusional themes. On account of the patient's complaint of daytime sedation, olanzapine was maintained at 20 mg at bedtime and augmented with oral haloperidol 5 mg twice daily. Haloperidol dose was increased to 10 mg twice daily on the third week of admission with significant improvement in auditory hallucinations and delusions. All liver enzymes were at baseline by the end of the third week of admission.

She was subsequently discharged on monthly haloperidol decanoate 100 mg IM and oral olanzapine 20 mg daily at bedtime. She was discharged on her 24th day of inpatient stay with normal liver enzymes and stable psychiatric symptomatology. Repeat liver enzymes and liver ultrasonography post discharge were normal at six weeks and remained within normal limits at outpatient follow-up three months following discharge.

## Discussion

Drug-induced liver injury and indeed clozapine-induced liver toxicity can be characterized by elevated serum alanine aminotransaminases, elevated ALP, and elevated bilirubin [1-3], as noted in our patient. Atypical antipsychotic treatment rarely results in severe liver toxicity [1,4]. First-generation antipsychotics such as chlorpromazine are more commonly associated with liver toxicity compared to second-generation antipsychotics [2,3]. Mild drug-induced liver injury is characterized by an elevated ALT, AST, and ALP without any elevation in bilirubin or PT/international normalized ratio (INR) abnormalities. Severe drug-induced liver injury may present with a hepatocellular pattern of liver injury characterized by elevated ALT/AST or with a cholestatic component in which ALP and serum bilirubin may also be elevated [1-3]. Hepatic steatosis may also be noted in antipsychotic-induced fatty liver. Liver sonography and abdominal CT findings may be characterized by hepatocellular inflammation and liver enlargement [5,6].

Asymptomatic increases in the levels of liver enzymes, especially ALT, are not uncommon with atypical antipsychotic treatment [3-5]. Laboratory and inflammatory changes on liver ultrasonography may appear after a few weeks of treatment and resolve spontaneously [5-7]. Liver inflammatory changes may persist as a result of fatty liver related to antipsychotic therapy [8]. Antipsychotic-induced liver injury may occur via hepatocellular, cholestatic, and mixed mechanisms [1,2,6]. Elevated transaminases (AST and ALT) suggest hepatocellular injury while elevated ALP and bilirubin may be indicators of cholestatic liver injury [1-3,6].

There have been reports of fatal cases following clozapine-induced fulminant hepatitis [5-7]. Liver enzymes up to three or four times above the upper limit of normal may be indicative of severe liver toxicity [1,2,5,7]. It is important to note that the magnitude of liver enzyme elevation does not correlate with the severity of the disease. Liver enzymes may be markedly elevated in asymptomatic patients and mildly elevated in very symptomatic patients [3].

The etiology of clozapine-induced hepatotoxicity is unknown presently [1,4,7]. It is unclear if clozapine-induced hepatotoxicity is idiosyncratic or dose-dependent [2,3,6], and there have been case reports of hepatotoxicity at low and high clozapine doses and varying clozapine treatment timelines. People of Asian ancestry may be predisposed to clozapine-induced hepatitis due to genetically reduced cytochrome p-450 1A2 levels with a resultant reduction in phase 1 hepatic clozapine metabolism [1,4,6].

Elevations in liver function tests in clozapine-treated patients are often temporary and asymptomatic [5]. Elevated liver enzymes have also been associated with fatty liver disease. Fatty liver and metabolic syndrome are common side effects of atypical antipsychotic treatment [5-7]. Up to a third of patients on initial clozapine dose titration may present with transient elevation in liver enzymes [1-4], while 60% of patients on long-term clozapine may develop a transient mild elevation in serum AST and ALT with spontaneous resolution. Over 15-30% of patients on clozapine may develop a marked increase in ALT and AST up to three times above normal levels requiring clozapine and other antipsychotic therapy cessation and work-up [1,4,7]. 

The most common clinical presentation of all drug-induced liver injuries is jaundice [2]. Other presenting symptoms may include malaise, pruritus, dark-colored urine, and pale stool [3,4]. Workup may show abnormal liver enzymes and elevated total and direct bilirubin. Fever, elevated autoimmune markers, and eosinophilia may be a pointer to an autoimmune etiology for hepatotoxicity [1,2]. Abnormal vitals, leukocytosis with neutrophilia, and elevated lactate levels might be a pointer to sepsis while eosinophilia, fever, and rash might be suggestive of a drug reaction with eosinophilia and systemic symptoms.

The pattern of liver enzyme elevation in clozapine-induced liver injury tends to be predominantly hepatocellular characterized by ALT and AST elevation. Cholestatic enzymes such as ALP and bilirubin may be elevated too. A mixed picture was seen in our patient as AST, ALT, ALP, and bilirubin were all moderately elevated. It is suggested that asymptomatic patients presenting with ALT three times above normal levels and ALP two to three times above the upper limit should be worked up for liver damage and antipsychotic medication(s) should be stopped [7].

Investigations required should include an abdominal ultrasound, ALT, AST, ALP, total bilirubin, albumin, INR, and PT [3,6,8]. Other important tests include a complete blood count (CBC) to identify an infectious etiology or drug rash with eosinophilia and systemic symptoms (DRESS) syndrome, hepatitis antibodies panel to rule out acute or chronic hepatitis, cytomegaloviral titers, and autoimmune antibodies such as antimitochondrial antibodies (AMA) and ds-DNA to identify an autoimmune etiology as possible differential diagnoses [2,3,7]. Liver imaging such as ultrasound and contrast-enhanced abdominal CT may show liver inflammation, ascites, gallstones, tumors, or cirrhosis [3-6]. Elevated total bilirubin and direct bilirubin over 20% of the total is in keeping with conjugated hyperbilirubinemia and might benefit from further workup. A liver biopsy may be helpful but is not required to make a diagnosis of clozapine hepatotoxicity [2].

Treatment is primary cessation of the offending medication. Cessation of clozapine is indicated in asymptomatic patients with ALT/AST levels up to three times of normal and ALP up to two times normal and in symptomatic patients who present with elevated liver enzymes [1-3].

Close outpatient monitoring and serial laboratory monitoring may be safe in asymptomatic patients following medication discontinuation [1,2]. Therapies such as ursodiol and antihistamines are effective for the outpatient treatment of pruritus in drug-induced liver disease. Inpatient medical admission may be warranted in asymptomatic patients with abnormal vitals and abnormal electrolytes, and in patients with symptoms consistent with acute liver failure such as encephalopathy and gastrointestinal bleeding [2].

Resolution of hepatotoxicity is typically expected with clozapine cessation [5-7]. Although there have been reports of fulminant liver failure and mortality despite clozapine cessation, long-term liver injury following clozapine cessation is unusual. Recommencing antipsychotic treatment depends on the patient’s psychiatric symptomatology. Treatment options following recovery include clozapine rechallenge in which the patient is recommenced on beginner doses of clozapine followed by a slower progressive clozapine dose titration [5,7,8]. Clozapine rechallenge is an option in patients following resolution of hepatotoxicity however a re-elevation of liver enzymes has been reported [5-7].

Another option is antipsychotic therapy switch [5,7,8]. Olanzapine is an alternative second-generation antipsychotic medication for patients with schizophrenia. Both olanzapine and haloperidol undergo phase two liver metabolism via glucuronidation, which is mostly preserved in hepatic disease [1,2]. Haloperidol is primarily metabolized in the liver via glucuronidation; olanzapine also predominantly undergoes phase two glucuronidation for its hepatic metabolism. Olanzapine is rarely associated with liver injury despite olanzapine being a cyt-1A2 substrate like clozapine and chlorpromazine [4,6]; hence, combination therapy of both olanzapine and haloperidol is an option for patients with refractory schizophrenia with poor response or intolerance of clozapine monotherapy [1,4].

Treatment-resistant schizophrenia is difficult to manage, and clozapine is often the last option. We proffer an alternate treatment modality which is a combination of haloperidol and olanzapine while acknowledging that olanzapine is metabolized by cytochrome p450 1A2 just like clozapine but is less likely to cause hepatotoxicity. Of note, antipsychotics like quetiapine and risperidone have been described as having a better hepatic safety profile compared to clozapine and olanzapine. Olanzapine received a higher consideration as the second-generation antipsychotic of choice in this patient since she presented with chronic psychotic symptomatology at baseline despite clozapine therapy and her psychiatric symptoms had rapidly worsened with the cessation of clozapine therapy, her BMI was also 36.2 on admission. Treatment should be on an individualized basis.

There are no clear guidelines with regard to how frequently liver function tests should be monitored in patients on clozapine [2,6]. Six-monthly liver enzyme monitoring for patients on clozapine has been suggested but there is no consensus for this. It is unclear if long-acting Injectable antipsychotics predispose more to hepatotoxicity compared to oral antipsychotics, especially in the setting of prior liver toxicity. Patients with alcohol use disorder, active liver disease, fatty liver associated with metabolic syndrome, elderly patients, and patients of Asian descent are more likely to benefit from increased frequency of liver enzyme monitoring [2,8]. Liver function tests should be ordered at baseline and are beneficial at the onset of symptoms such as jaundice, pruritus, and fever. Nonspecific symptoms such as confusion, malaise, and fatigue while on clozapine antipsychotic therapy may also warrant liver panel testing [4-6].

## Conclusions

Hepatotoxicity is unusual in clozapine patients and clinicians must be on the lookout for signs and symptoms of hepatic injury during the course of clozapine therapy regardless of duration of treatment. Clozapine-induced hepatotoxicity may be idiosyncratic or dose-related. Predisposing factors for hepatotoxicity during clozapine therapy includes genetic variation in cytochrome p-450 1A2 levels, age, drug-drug interactions and chronic liver disease. Geriatric patients, patients with preexisting liver disease and alcohol use disorder might benefit from increased frequency of liver enzyme monitoring while on clozapine. Complaints of fatigue, pruritus and jaundice while on clozapine therapy also warrants liver enzyme testing. A combination of first and second-generation antipsychotic treatment such as an olanzapine-haloperidol combination may be an effective alternative treatment option for treatment-refractory patients who develop hepatotoxicity while on clozapine. 
